# Meteorological Register for November, 1816

**Published:** 1817-01

**Authors:** John Bailey

**Affiliations:** Surgeon, &c.


### Meteorological Register for November, 1816.

*Kept at Harwich, in Essex.*

By JOHN BAILEY, Surgeon, &c.

| Moon's Age. | Atmospherical Pressure. Morn. | Ev. | Temp. | Wind. | Remarks. | General Statement of Diseases. |
|---|---|---|---|---|---|---|
| 1 | 29 3 | 29 3 | 54 | S | Rain | |
| 2 | 3½ | 3 | 51 | S | Ditto. Foggy | |
| 3 | 3½ | 6 | 51 | S | ditto | |
| 4 | 6 | 7 | 50 | E | ditto | Pneumonia |
| ○ 5 | 6½ | 7 | 51 | E | ditto | |
| 6 | 4 | 3 | 48 | | ditto | Catarrhus |
| 7 | 3 | | 42 | NW | ditto | |
| 8 | 4½ | 4½ | 31 | NW | Rain. Windy | Intermittentes |
| 9 | 28 9 | 29 8 | 32 | SW | Snow. Gale of w. | |
| 10 | 29 2 | 5 | 32 | N | ditto | Varicella |
| 11 | 6 | 6 | 28 | W | ditto | |
| ☽ 12 | 1 | 6 | 42 | N | ditto | |
| 13 | 6 | 6 | 50 | W | ditto | |
| 14 | 6 | 6 | 43 | NW | ditto | |
| 15 | 6 | 4 | 32 | NW | Snow. Ditto | |
| 16 | 6½ | 8 | 33 | N | ditto | |
| 17 | 9½ | 9½ | 33 | N | | |
| 18 | 7 | 7 | 42 | SW | Much rain | |
| ● 19 | 7 | 7 | 43 | | Sun eclipsed | |
| 20 | 9 | 30 | 46 | SW | Fine and clear | |
| 21 | 30 | 29 5½ | 43 | SE | Dull | |
| 22 | 29 8 | 7 | 37 | E | | |
| 23 | 7 | 8 | 34 | E | | |
| 24 | 8½ | 8½ | 28 | NW | | |
| 25 | 9 | 9 | 32 | SE | | |
| ☾ 26 | 9 | 30 ½ | 42 | | Rain. Thick fog | |
| 27 | 30 ½ | 2 | 27 | SW | Foggy | |
| 28 | 2 | 2 | 40 | SW | ditto | |
| 29 | 3 | 3½ | 38 | NW | | |
| 30 | 4½ | 5 | 35 | N | Fine and clear | |

Catarrhal affections have been common, as they usually are at this season of the year; a few cases put on a serious character, but terminated favourably. Much rain came to theearth in the first ten days of this month; intermittents followed: but the continuance afterwards of brisk gales dried away the wet, and these ailments gave way readily to the milder forms of tonics.

